# Sunstroke

**Published:** 1879-08-20

**Authors:** 


### *SUNSTROKE—INSOLATION.*

The season of year is upon us when we may expect much suffering and not a few deaths from the effects of heat. Last summer, in St. Louis, there were many cases, but thanks to a more rational plan of treatment than was in vogue a few years ago, fatal results were rare. The affection is to be dreaded, not only because it is occasionally fatal, but also on account of the sad sequelæ that so often follow it.

It is a disease of the nervous system induced by heat, usually following exposure to the direct rays of the sun, from which it takes its name of "sunstroke;" but as all cases are not due to such exposure, this designation is a misnomer. "Calenture" would be preferable (from *calere*, to be hot), for a continued high temperature, even indoors, will produce it, as is seen in the effect on persons working in the confined, heated atmosphere of foundries, sugar-refineries, kitchens, laundries, on board vessels, and in barracks. Previous exhaustion of the system, from whatever cause, predisposes to it. Laborers suffer mostly, especially if of intemperate habit or of debilitated systems. In times past soldiers have suffered greatly; this was exemplified in our own late war. Men from the North, not accustomed to the extreme heat of the South, succumbed while on march frequently. In a short march of ten miles, on an extremely hot day, we have witnessed one-tenth of the men so affected. The heavy and tight clothing, together with the weight of the accoutrements, played an important part as factor in producing the disease. More judgment is at present displayed in the dressing of troops serving in hot climates, so that sunstroke is less frequent among them than formerly. The lamented Havelock hoped to prevent some of the evil effects of the direct rays of the sun by having the men wear white linen hoods with long capes.

Exposure to long-continued heat may be borne with impunity if the functions of the skin and bowels are active, but in cases of sunstroke the functions of the skin cease; thus the bodily temperature necessarily runs up, and the blood becomes impure; the bowels being constipated adds to the blood impurity. The frequent micturition is due not so much to renal activity as to the irritable urine.

The duration of the disease may vary—being fatal in a few minutes or in forty-eight hours. It is not the rule, however, that it ends in death; but with recovery, if incomplete, come sequelæ, such as impairment of the intellect, distressing headache, amaurosis and other evidences of cerebral trouble, such as twitching of the muscles of the forearm and hand.

The symptoms vary with the cause of the attack. In the cardiac variety the onset is sudden, with scarcely premonitory symptoms. The patient falls, pants for breath, and may at once expire from syncope. In the cerebro-spinal variety the impending danger is indicated by heat and dryness of skin, temperature rising possibly as high as 107° F., vertigo, pain in the head (not constant), congestion of eyes, tired feeling, nausea, frequent desire to micturate, in severe cases even delirium. If the patient is conscious of the approaching attack, he may lie down and pass into a comatose condition. If the case progresses unfavorably the pupils contract—with insensibility to light, the symptoms intensify, with possible convulsions.

The attack need not be mistaken for apoplexy, in which the pulse is slow, full, possibly intermitting; breathing slow, irregular, explosive; pupils dilated, irregularly; skin cool, moist. In sunstroke the pulse is quick and sharp; breathing rapid, noisy, but not explosive; pupils contracted, and conjunctiva injected; skin hot and dry, except in a few of the cardiac cases.

The superheating of the blood has a depressing effect on the nervous centres; thus the cause of the symptoms as above given, and thus too, the index to treatment.

Prolonged insensibility, continued heat of skin, increasing congestion of the eyes, irregular and failing action of the heart, and lividity of the extremities, are unfavorable signs. Death may be preceded by convulsions.

After death the blood is found fluid; possible congestion of the cerebral vessels, and congestion of the lungs with, in the cardiac variety, distention of the right heart.

Protection from the sun, the wearing of light and loose clothing, avoidance of stimulants and of prolonged exertion in a close, heated atmosphere, are all prophylactic.

In the treatment, blood-letting was formerly resorted to, never now, for we have not to do with cerebral apoplexy. The results of therapeutic measures at present are gratifying, where formerly they were too often serious. The patient should be placed in the shadiest and coolest spot to be found, stripped and douched with cold water over head and chest, followed up or repeated until the temperature of the skin is reduced, and the respiration more regular. If the skin is not hot, the use of the water may be limited to sprinkling the face and chest. Ice may be used to lessen the temperature of the water. Empty the bowels by enema, afterward injections of ice-water may be used. Apply ammonia cautiously to the nostrils. If the skin refuses to act, we would advise the fluid extract of jaborandi in 20-minim doses, repeated every half hour until three or four doses has been taken, to induce sweating. Bromide of potassium in ten-grain doses every twenty minutes, well diluted, should be given, and if necessary, by enema, in which cases twenty grains may be administered. When exhaustion is imminent and the pulse weak, stimulants must be used by either the stomach or rectum. In these latter cases the cold douche must be employed carefully, and only for its effect as a shock.

If sensibility be not restored, a blister to the nucha should be applied. Dyspnea, when marked, may be relieved by application of dry cups to the thorax behind and in front. If convulsions set in, chloroform inhalations may be resorted to. After consciousness is restored, sinapisms may be applied to the nucha and to the mastoid regions, and the bromide should be continued for many days. Quietness should be enjoined, both of mind and body, and if possible, removal to a cooler climate. Regular habits should be insisted upon, without excesses.

Persistent headache frequently follows sunstroke as a sequel; in its treatment counter-irritation to the nucha, long continued, with a course of iodide of potassium, is often followed by benefit. A permanent residence in a cool climate, or at least removal to such during the hot season of the year, is of great benefit. Bathing of the skin, frictions, open-air exercise, regulation of all the functions, especially of the stomach and bowels, are quite essential.—*St. Louis Couriet of Medicine.*

